# Characterization of the equine 2'-5' oligoadenylate synthetase 1 (*OAS1*) and ribonuclease L (*RNASEL*) innate immunity genes

**DOI:** 10.1186/1471-2164-8-313

**Published:** 2007-09-07

**Authors:** Jonathan J Rios, Andrey A Perelygin, Maureen T Long, Teri L Lear, Andrey A Zharkikh, Margo A Brinton, David L Adelson

**Affiliations:** 1 Department of Animal Science, Texas A&M University, 2471 TAMU, College Station, Texas 77843, USA; 2 Biology Department, Georgia State University, 24 Peachtree Center Ave., Atlanta, Georgia 30302, USA; 3 College of Veterinary Medicine, University of Florida, 2015 SW 16th Ave., Gainesville, Florida 32608, USA; 4 Department of Veterinary Science, University of Kentucky, 108 Maxwell H. Gluck Equine Research Center, Lexington, Kentucky, 40546, USA; 5 Bioinformatics Department, Myriad Genetics, Inc., 320 Wakara Way, Salt Lake City, UT, 84108, USA; 6 School of Molecular and Biomedical Science, University of Adelaide, SA 5005, Australia

## Abstract

**Background:**

The mammalian OAS/RNASEL pathway plays an important role in antiviral host defense. A premature stop-codon within the murine *Oas1b *gene results in the increased susceptibility of mice to a number of flaviviruses, including West Nile virus (WNV). Mutations in either the *OAS1 *or *RNASEL *genes may also modulate the outcome of WNV-induced disease or other viral infections in horses. Polymorphisms in the human OAS gene cluster have been previously utilized for case-control analysis of virus-induced disease in humans. No polymorphisms have yet been identified in either the equine *OAS1 *or *RNASEL *genes for use in similar case-control studies.

**Results:**

Genomic sequence for equine *OAS1 *was obtained from a contig assembly generated from a shotgun subclone library of CHORI-241 BAC 100I10. Specific amplification of regions of the *OAS1 *gene from 13 horses of various breeds identified 33 single nucleotide polymorphisms (SNP) and two microsatellites. *RNASEL *cDNA sequences were determined for 8 mammals and utilized in a phylogenetic analysis. The chromosomal location of the *RNASEL *gene was assigned by FISH to ECA5p17-p16 using two selected CHORI-241 BAC clones. The horse genomic *RNASEL *sequence was assembled. Specific amplification of regions of the *RNASEL *gene from 13 horses identified 31 SNPs.

**Conclusion:**

In this report, two dinucleotide microsatellites and 64 single nucleotide polymorphisms within the equine *OAS1 *and *RNASEL *genes were identified. These polymorphisms are the first to be reported for these genes and will facilitate future case-control studies of horse susceptibility to infectious diseases.

## Background

The innate immune responses are the first line of host defense against a virus infection. An important component of the intracellular antiviral response is mediated by the 2'-5' oligoadenylate synthetase (OAS)/ribonuclease L (RNase L) pathway. OAS genes are interferon-inducible and activated by binding to double-stranded RNA (dsRNA). dsRNA, present in virus infected cells, activates OAS proteins to catalyze the oligomerization of ATP to form 2',5' -linked oligoadenylate chains (pppA(2'p5'A)_n_) [1-3]. Originally discovered as a low molecular weight inhibitor of protein synthesis, pppA(2'p5'A)_n _induces the activation of the latent endoribonuclease, RNase L, which degrades both cellular and viral RNA in a non-preferential manner [1,4-6]. The OAS/RNASEL pathway has also been implicated in the induction of apoptosis [7-11].

The murine flavivirus resistance gene, *Flv*, was positionally cloned and identified as *Oas1b *[12]. A cDNA sequence comparison among susceptible and resistant strains of mice identified a single nucleotide substitution that causes a premature stop codon in the Oas1b transcripts of susceptible mice [12,13].

The human OAS gene cluster, consisting of genes *OAS1*, *OAS3 *and *OAS2*, is located on chromosome 12q24.2 [14]. The small synthetases are transcribed from the *OAS1 *gene while the medium and large synthetases are encoded by the *OAS2 *and *OAS3 *genes, respectively. Alternative splicing was previously reported in both *OAS1 *and *OAS2 *transcripts [15,16]. For example, the human *OAS1 *transcript E16 corresponds to the p42 protein, which is translated from a 1.6 kilobase (kb) mRNA, while the alternatively spliced E18 transcript encoding the p46 protein is about 1.8 kb [17]. Both p42 and p46 proteins are identical in their first 346 N-terminal amino acids but differ at the C-terminus [18]. Variations in the human *OAS1 *gene that may be relevant to the outcome of virus infections have been reported [19-23].

The human *RNASEL *gene maps to chromosome 1q25 [24]. The 741 amino acid, 83,539 Dalton protein is translated from a ~2.8 kb transcript [25,26]. The RNase L protein consists of three domains: 1) an N-terminal domain of ankyrin repeats with P-loop motifs between the seventh and eighth repeat, 2) a serine/threonine protein kinase domain, and 3) a C-terminal ribonuclease domain [27]. RNase L activation requires binding of a single 2-5A molecule to the N-terminal ankyrin repeats 2–4 [28,29]. 2-5A binding reverses the naturally repressive state of the RNase L ankyrin repeats, ultimately producing a functional homodimer with ribonuclease activity [27,29-31].

Previously, the equine OAS gene cluster was mapped to horse (*Equus caballus; ECA*) chromosome 8p15 and shown to have an organization similar to that in the human genome: *OAS1*-*OAS3*-*OAS2 *[32]. Two clones were identified from segment 1 of the CHORI-241 equine BAC library, 77F4 (~200 kb) and 100I10 (~130 kb), that contain complete *OAS1 *and *OAS3 *sequences. BAC clone 77F4 also contains nine 5'-terminal exons of *OAS2 *[32].

In this report, a subclone library generated from CHORI-241 BAC 100I10 was sequenced and then used to construct a contig assembly spanning the *OAS1 *gene. The equine *RNASEL *gene was identified in multiple BAC clones of the CHORI-241 library and was FISH mapped on metaphase spreads to ECA5p17-p16. Equine *RNASEL g*enomic sequence was obtained from BAC clone 159N12 and an assembly similar to that for *OAS1 *was constructed. Full-length *RNASEL *cDNA from 8 species were determined and compared in a phylogenetic analysis. Re-sequencing of genomic DNA from multiple horses of different breeds identified a total of 64 SNPs and 2 microsatellites within the *OAS1 *and *RNASEL *genes.

## Results

### BAC 100I10 sequencing and *OAS1 *contig assembly

A shotgun subclone library was constructed from sheared fragments of CHORI-241 BAC 100I10. Nine hundred sub-clones were bi-directionally sequenced, resulting in 513,390 bases with quality scores > 15, providing 3.95X coverage. The individual chromatogram files were analyzed by Phred, Phrap and Consed [33-37] and individual contigs were scaffolded on the human genome sequence using BLAST. The scaffold was further validated by the addition of multiple sequences from TraceDB [38] retrieved via BLAST searches using full length equine *OAS1 *mRNA [GenBank: AY321355]. The scaffold contained four genomic contigs spanning a substantial part of the equine *OAS1 *gene, including 4.5 kb of promoter sequence upstream of exon 1 and 1.6 kb of sequence downstream of exon 6, and was submitted to GenBank under accession number DQ536887. The genomic assembly also included sequence for the downstream equine *OAS3 *gene as well as an upstream gene orthologous to human *RPH3A *(data not shown). This assembly completely overlaps two whole genome shotgun sequences, AAWR01028567 (55,475 bp) and AAWR01028568 (31,407 bp), that were recently submitted to GenBank from the Broad Institute.

### Identification of *OAS1 *microsatellites

**Figure 1:**
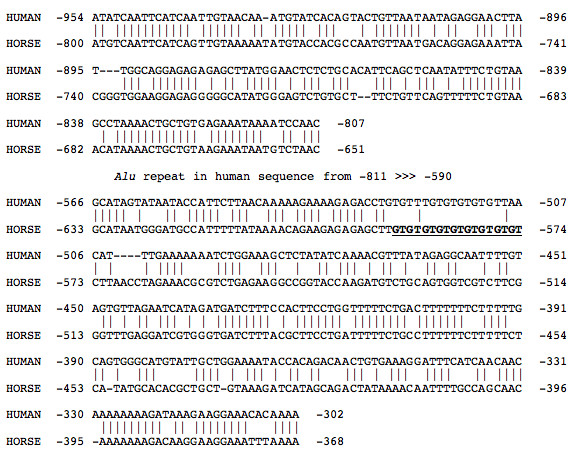
**Local alignment of human and horse *OAS1 *promoters**. BLAST2 alignment of the 1000 bp upstream of the transcription start for human *OAS1 *and equine *OAS1 *genes. The following BLAST parameters were used: a mismatch penalty of -1 and word size of 7. Lower case masking of repeats was used. The alignment shows that the sequence from ~800 bp to ~-350 bp in the horse promoter is similar to a region of the human promoter interrupted by a 200 bp *Alu *repeat (~-811 bp ~-590 bp). The horse microsatellite is shown in underlined bold and corresponds to a smaller dinucleotide repeat in the human sequence. Numbering shown in the alignments is from the translation ATG start sites.

The genomic sequence assembly identified two microsatellites, one located within the promoter and the other downstream of exon 6. The promoter GT-microsatellite is located 575 bp upstream of the ATG translation initiation site. A shorter GT-microsatellite is in the same relative position in the human *OAS1 *promoter and the flanking regions were well conserved between the two sequences (Figure 1). This microsatellite may affect the functions of flanking regulatory elements. Sequencing the OAS1 promoter regions of 13 horses established that this promoter microsatellite is polymorphic in length. The second polymorphic microsatellite was a GT-dinucleotide repeat located 43 bp downstream of exon 6 within the 3' UTR. It has previously been reported that a 3' UTR microsatellite can alter the level of synthesis of a mRNA. [39].

### *OAS1 *SNP identification

The assembled *OAS1 *scaffold was aligned to the full length, 1.6 kb cDNA equine transcript [GenBank: AY321355] to delineate individual exons and flanking intron sequences from the genomic contigs. Genomic primers were designed within flanking intron sequences as well as for the proximal promoter (Table 1).

**Table 1:** Primers used to detect equine polymorphisms

**Gene**	**Region**	**Forward Primer**	**Reverse Primer**
OAS1	Promoter	AACCCACAGAATAAACACCACA	GTCGATGGCTTCTCGGAC
	Exon 1	CCAGACTCAGGCAACGTAAG	GTTTTGCTCTCTCCCTTCCT
	Exon 2	GTGATTGTTGTCTGGTGATGG	AAACTGTGGGAGATTTCTGCT
	Exon 3	GTAACTTGGTTGTGTCCGTGG	AGACTGGATGGAGGGCCATA
	Exon 4	AGCGTGAAAACCACCACAGA	TCCCACATCCTCCATTTCC
	Exon 5	CACTGGGCTGGTCCTCCT	CCTCCAAACGGGGTCAAA
	Exon 6	GCAGGTGGCACGTCACAG	GGCACTGTGCCCTGAAGTTAA

RNASEL	Exon 1	CATCTCCCTTCTCCGTCCTCG	TGCAATGGATGAGTCCTGGT
	Exon 2	CAAAGTACTTCTCTCATCCCCAG	TCCGAAGAGCATGGAACAAA
	Exon 2	AAGATCCTCCTTGATGAGATGG	GGCCTTTCCTATCTGCAATG
	Exon 3	AAATGTAAGTCCTGCTCTTGGC	CAAGCAACTCCACACCAACC
	Exon 4	CTCGTAGCCTGCACCACAC	CACGGTAGATCGCGGAACTT
	Exon 5	CCATGTTAATTCTCTCATCTTCAG	TCTCTCACCTCTTGGTAGGGC
	Exon 6	GCTCCTACATTTTTGCGTAATG	GTTCTTCCCATCAAATAGCAGA
	Exon 7	ATCTCTGGAACCGGGTGCT	CACTACCAAATGGCCCTGAG
	Exon 7	CTCTGGGTGGCTCATTCATT	TCCCAGCTCTTCCCATTACA

Forward and reverse primers for amplification of the *OAS1 *promoter and individual exons of equine *OAS1 *and *RNASEL*.

Sequence data obtained from the screening population and from CHORI BAC 100I10 were analyzed using Phred, Phrap and Consed programs [33-37]. Both visual analysis of the chromatogram data to identify heterozygotes and computer analysis using the Consed visualization tool identified 33 single nucleotide substitutions within the proximal promoter and exons of *OAS1 *(Table 2). Of these, 11 were within coding regions, 9 within non-coding regions and the remaining 13 within the proximal promoter upstream of exon 1. Four of the 9 non-coding polymorphisms were located within the 5' and 3' untranslated regions (UTR). Of the 11 coding polymorphisms, 4 were synonymous and 7 were non-synonymous. Five of the 7 non-synonymous SNPs resulted in substitutions of amino acids with different properties. Interestingly, the amino acids encoded by the major alleles of 4 of the 7 non-synonymous mutations were identical to the corresponding amino acids in the human OAS1 protein [UniProtKB: P00973]. The genotypes of each individual were used to identify potential haplotypes within equine *OAS1 *using PHASE v2.1 software [40,41]. Only those SNPs verified within multiple individuals were used for the haplotype analysis (minor allele frequency = 0.08). The best reconstruction produced 15 haplotypes from the 33 diallelic SNPs (Table 3). The polymorphic microsatellites were not included in the analysis.

**Table 2:** Equine *OAS1 *single nucleotide polymorphisms and microsatellites

**Region**	**Accession **DQ536887	**Alleles**	**Residue**	**Amino Acid Type**	**Frequency**	**Polymorphism**
-	3640	C	-	-	0.25	Transition
		T			0.75	
-	3687	G	-	-	0.65	Transversion
		T			0.35	
-	3718	A	-	-	0.65	Transition
		G			0.35	
-	3724	C	-	-	0.65	Transition
		T			0.35	
-	3825	C	-	-	0.35	Transition
		T			0.65	
-	3830	A	-	-	0.85	Transversion
		T			0.15	
-	3973	C	-	-	0.65	Transition
		T			0.35	
-	4032–4063	-	-	-	-	GT repeat
-	4234	C	-	-	0.65	Transition
		T			0.35	
-	4333	C	-	-	0.35	Transition
		T			0.65	
-	4455	C	-	-	0.08	Transversion
		G			0.92	
-	4487	C	-	-	0.88	Transition
		T			0.12	
-	4501	C	-	-	0.65	Transition
		T			0.35	
-	4531	A	-	-	0.08	Transition
		G			0.92	
5' UTR	4598	C	-	-	0.65	Transversion
		G			0.35	
5' UTR	4625	A	-	-	0.35	Transversion
		C			0.65	
Exon 1	4690	A	18Tyr	Uncharged Polar	0.85	Transition
		G	18Cys	Uncharged Polar	0.15	
Exon 1	4783	C	49Ala	Nonpolar	0.35	Transition
		T	49Val	Nonpolar	0.65	
Intron 1	5609	C	-	-	0.67	Transition
		T			0.33	
Exon 2	5701	C	77Leu	Nonpolar	0.64	Transition
		T	77Leu		0.36	
Exon 2	5743	C	91Phe	Nonpolar	0.33	Transition
		T	91Phe		0.67	
Exon 2	5765	A	99Lys	Basic Polar	0.65	Transition
		G	99Glu	Acidic Polar	0.35	
Exon 2	5776	A	102Arg	Basic Polar	0.38	Transition
		G	102Arg		0.62	
Exon 2	5786	A	106Lys	Basic Polar	0.38	Transition
		G	106Glu	Acidic Polar	0.62	
Exon 2	5920	G	150Pro	Nonpolar	0.08	Transversion
		T	150Pro		0.92	
Exon 3	9374	C	209Arg	Basic Polar	0.85	Transition
		T	209Cys	Uncharged Polar	0.15	
Exon 4	12714	C	264Asn	Uncharged Polar	0.59	Transversion
		G	264Lys	Basic Polar	0.41	
Intron 4	12810	C	-	-	0.64	Transition
		T			0.36	
Intron 4	12853	A	-	-	0.55	Transition
		G			0.45	
Intron 5	13628	A	-	-	0.55	Transversion
		T			0.45	
Intron 5	13649	C	-	-	0.46	Transversion
		G			0.54	
Exon 6	15320	C	370Arg	Basic Polar	0.75	Transition
		T	370Trp	Nonpolar	0.25	
3' UTR	15410	G	-	-	0.62	Transversion
		T			0.38	
3' UTR	15537	G	-	-	0.19	Transversion
		T			0.81	
-	15798–15855	-	-	-	-	GT repeat

SNPs and microsatellites identified from sequencing the *OAS1 *proximal promoter and exons from 13 equine individuals and CHORI BAC 100I10. Positions are identified from the genomic consensus sequence submitted to GenBank [GenBank: DQ536887].

**Table 3:** Equine *OAS1 *and *RNASEL *haplotypes

**Gene**	**Haplotype Sequence**	**Count Frequency**
OAS1	CTGTCATTCGCTGGAACCCTAGGTCGTAACCTT	0.08
	CTGTCTTTCGCTGGAACCCTAGGTCCCATGCGT	0.04
	CTGTCTTTCGCTGGAACCCTAGGTCCCGTGCGT	0.08
	CTGTCTTTCGCTGGAACCCTAAGTCCCGTGCGT	0.04
	TGACTACCTGCCGCCATCCTAGGTCGTAACCTT	0.19
	TGACTACCTGCCGCCATCCTAGGTCGTAAGCTT	0.04
	TGACTACCTGCCGCCATCCTAGGTCGCAACCTT	0.04
	TGACTACCTGCCGCCATCCTAGGGCGTAACCTT	0.04
	TGACTACCTGCCGCCATTTCGAATCCCGTGTGT	0.11
	TGACTACCTGCCGCCGTTTCGAATCCCAACCGT	0.08
	TGACTACCTGTCGCCGTTTCGAATCCCGACCGG	0.08
	TGACTACCTCCCACCATTTCGAATCCCGTGTGT	0.04
	TGACTACCTCCCACCATTTCGAAGCCCGTGTGT	0.04
	TTGTCATTCGCTGGAACCCTAGGTTCCGTGTGG	0.08
	TTGTCATTCGTTGGCACCCTAGGTTCCGTGTGG	0.04

RNASEL	GACTGCAAAGGGAGCGCTGGGCAGTTTCTTT	0.07
	GATCGCAAGGACGGCGCTGGGCACACCCCCC	0.04
	GATCGCAAGGACGGCGCTGGGCACACCTCCC	0.12
	GATCTCACAGGGAGCGCTGGGCAGATTCTTC	0.12
	GATCTCACAGGGAATAACAAGTGGACCCCCC	0.12
	GATCTAGAGGACGGCGCTAGGCAGATTCTTC	0.07
	GCCCGCAAGGGCGGCGCTAGGCAGATTCTTC	0.23
	GCCCGAAAGGGGAATAACAAGTAGATTCTTC	0.07
	GCCCGAAAGTGGAGCGCTGGGCAGTTTCTTT	0.04
	CCCCGAAAGTGGAGCGCTAGACAGACCCCCC	0.12

Haplotypes were assembled using PHASE v2.1 [40, 41] under default settings. The haplotypes identified from the best reconstruction are shown with their corresponding frequencies among the 13 horses screened for both *OAS1 *and *RNASEL *SNPs. Both *OAS1 *microsatellites were omitted from the haplotype reconstruction.

### Assembling full-length *RNASEL *mRNA sequences of cattle, dog, horse, cat, domestic pig, Guinea pig, elephant and opossum

A limited number of mammalian *RNASEL *mRNA sequences were previously deposited to GenBank and some of these sequences were predicted from whole genome annotations. However, this GenBank information was not sufficient to identify evolutionarily conserved regions in mammalian *RNASEL *sequences that could be used to design PCR primers to amplify equine *RNASEL *fragments. The predicted sequences of cattle [GenBank: XM_597290] and dog [GenBank: XM_547430] *RNASEL *ORFs were amplified from commercial cDNA (BioChain, Hayward, CA), directly sequenced and extended to full-length cDNA sequences by DNA walking. The full-length cattle and dog *RNASEL *sequences were submitted to GenBank under accession numbers DQ497162 and DQ497163, respectively. These two sequences as well as the human full-length *RNASEL *sequence NM_021133 were aligned and degenerate primers were designed from conserved regions (Table 4) and used to amplify the middle portions of equine *RNASEL *cDNA. This partial sequence was extended to the full-length sequence by DNA walking and submitted to GenBank under accession number DQ497159.

**Table 4:** *RNASEL *primers

**Species**	**Forward primer**	**Reverse primer**
Cat	CAGGCATCCAGAAGGGAGAC	CAGAGGCAGCCAATCTCTCC
Cattle/Dog	GCTGGTCACCTTTGCATAATGC	CCCAACTCCAAAAGAAGGGATG
Domestic pig	ATGGAGACCAAGCGCCATAACA	TGTTCTCCCAAGTTCCGGATGA
Elephant	ATATCCCTACTAGCCTGACGAG	TTGCCTTGACACCCCCAATTCT
	AGCTGTAGGATGTAACTCTCACT	GATTAGAGGAACCACTGAGAGG
	GCGGTACCTCATTGTGGTTTTG	CCTCTGTATCTTCATGGTCTGG
	TGCCTTTGAATTGTGGTGTTGGT	CCATGTGGTGGATTCATTATAGG
	GTTGAGGTGTCAGGATCTGCAT	GGGGTAACACTGGAACTGTTTC
Guinea pig	TAATGGTCTGGACCATTCCTCC	GTTTGAGGAAAGTGCCTTGCGT
Horse	TTCACRGCYTTCATGGAAGC	CYTTKATCAAAATCTGCCAG

Primers used to assemble the full-length *RNASEL *mRNA sequences of cattle, dog, horse, cat, domestic pig, Guinea pig, elephant and opossum.

Several additional mammalian *RNASEL *sequences were also determined and subsequently used to perform a phylogenetic analysis. The GenBank feline Whole Genome Sequence (WGS) database was searched with the canine *RNASEL *sequence [GenBank: DQ497163]. Four genomic contigs, AANG01026257, AANG01026302, AANG01630549 and AANG01026248, were detected. These contigs contain the first, second and third, as well as the fifth and sixth feline *RNASEL *coding exons, respectively. No contigs containing the fourth coding exon of the feline *RNASEL *gene were found in GenBank. Two primers were designed based on the 3'- end AANG01026302 sequence and the 5'-end AANG01630549 sequence (Table 4) and used to amplify and sequence this region from a commercial cat genomic DNA (Novagen, Madison, Wisconsin). The sequence of this exon was submitted to GenBank under accession number EF062998. Using this sequence as well as the other exon sequences derived from GenBank (see above), the predicted full-length mRNA sequence of the feline *RNASEL *gene was assembled.

The TIGR porcine database [42] was searched using the cattle sequence [GenBank: DQ497162] and five partial *RNASEL *sequences were found. The TC212507 and TC212872 sequences correspond to the 5'-end of porcine *RNASEL *mRNA, while the TC218317, TC237301, and TC236970 sequences represent the 3'-end. An additional 5'-end cDNA sequence, 20060611S-038813, was detected in the Pig EST Data Explorer [43]. A pair of primers were designed based on the partial sequence (Table 4) and used to amplify pooled cDNA (kindly provided by Dr. Jonathan E. Beever, University of Illinois at Urbana-Champaign). The middle portions of the porcine *RNASEL *cDNA were directly sequenced. The partial sequence was then extended to the full-length sequence by DNA walking and submitted to GenBank under accession number DQ497160.

The GenBank Guinea Pig whole genome sequence database was searched using both mouse [GenBank: NM_011882] and rat [GenBank: NM_182673] full-length *RNASEL *sequences. Two Guinea pig sequences, AAKN01052053 and AAKN01424676, showed significant similarity to the 5' and 3' regions of the rodent *RNASEL *sequences, respectively. These two sequences were used to design primers (Table 4) to amplify commercial cDNA (BioChain, Hayward, CA) and directly sequence the middle portions of Guinea pig *RNASEL *cDNA. This partial sequence was extended to the full-length sequence by DNA walking, and submitted to GenBank under accession number DQ497161.

Cattle, dog, horse and pig *RNASEL *sequences were used to search the GenBank elephant genome trace archive using the discontiguous Mega BLAST program. The same sequences were also used to search the GenBank elephant whole genome sequence database using the BLASTN program. The sequences for all potential exons of the elephant *RNASEL *gene were identified. Based on these sequences, five primer pairs (Table 4) were designed to amplify genomic DNA (kindly provided by Drs. Alfred L. Roca and Stephen J. O'Brien, National Cancer Institute) and directly sequence each of the elephant *RNASEL *exons. The resulting sequence was submitted to GenBank under accession number DQ497164.

The *RNASEL *ORF sequence of the laboratory opossum (*Monodelphis domestica*) was predicted by searching the UCSC genome browser [44] using the BLAT program. No sequence traces similar to *RNASEL *were detected in frog (*Xenopus tropicalis*) or several fish species (*Danio rerio*, *Takifugu rubripes *and *Tetraodon nigroviridis*).

### Phylogenetic analysis of vertebrate *RNASEL *gene sequences

**Figure 2:**
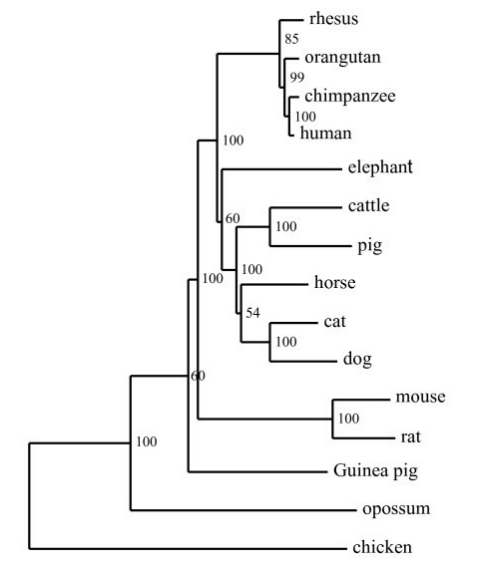
**Phylogenetic tree of *RNASEL *genes**. *RNASEL *ORF sequences from 15 vertebrate species were aligned and the njtree program was used for tree construction.

Only sequences of human and mouse RNASEL genes were previously reported [26]. Sequences of orthologous rat (GenBank: AY262823) and chicken (GenBank: AM0492248) genes were recently submitted to GenBank but have not been reported in any publications. In addition, annotations of chimpanzee, orangutan and rhesus macaque genomes using a GNOMON method resulted in predicted RNASEL sequences in these three species. Primate, rodent and avian RNASEL sequences were downloaded from GenBank and aligned to orthologous sequences described above to build a phylogenetic tree (Figure 2). Rodents showed the highest rate of nucleotide substitutions, while primates showed the lowest rate of evolution. Evolution rates were found to be fairly uniform in the three different RNase L domains: ankyrin repeats, serine/threonine protein kinase domain, and ribonuclease domain. The percent identity between the RNASEL ORFs of horse and the other species compared is shown in Table 5.

**Table 5:** Lengths of coding exons (bp) within the ORFs of vertebrate *RNASEL *genes and percent identity between horse and other species *RNASEL *ORFs

**Species**	**Exon A**	**Exon B**	**Exon C**	**Exon D**	**Exon E**	**Exon F**	**Identity**
Horse	1480	86	206	133	134	130	100.0
Cat	1477	86	206	133	134	139	83.0
Dog	1477	86	206	133	134	139	81.1
Cattle	1474	86	206	130	131	145	79.2
Elephant	1510	86	206	133	137	187	75.1
Human	1480	86	206	133	134	187	81.9
Chimpanzee	1480	86	206	133	134	187	79.7
Orangutan	1480	86	206	133	134	187	81.2
Rhesus	1480	86	206	133	134	187	79.7
Mouse	1474	86	206	133	137	172	66.3
Rat	1489	86	206	133	131	172	65.5
Guinea Pig	1462	86	206	133	134	187	69.7
Opossum	1453	86	206	129	131	139	56.5
Chicken	1402	89	191	124	122	136	37.3

### Assignment of the *RNASEL *gene to horse chromosome ECA5p17-p16

**Figure 3:**
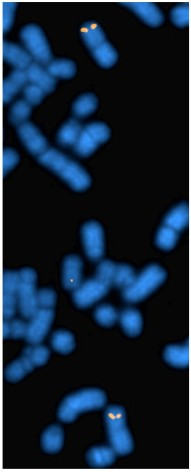
**FISH mapping of equine *RNASEL***. FISH map position ECA5p17-p16 of horse *RNASEL *gene (orange) on DAPI counterstained metaphase chromosomes (blue).

The horse CHORI-241 BAC library was searched with a probe derived from the partial equine *RNASEL *cDNA fragment. Twelve positive clones were identified and two of them, 108P15 and 189I19, were FISH mapped to assign the *RNASEL *gene to the horse chromosomal location ECA5p17-p16 (Figure 3).

### Exon/intron structures of vertebrate *RNASEL *genes

Partial sequence for the equine *RNASEL *gene was obtained by sequencing PCR fragments of BAC 159N12. The mRNA sequence [GenBank: DQ497159] was used as a reference for determining intron/exon junctions. Sufficient genomic sequence was obtained to build a scaffold as described for the equine *OAS1 *gene. The scaffold was verified using sequences from TraceDB [38] and submitted to GenBank under accession number EF070193. This scaffold completely overlaps the whole genome shotgun sequence AAWR01030439 (193510 bp) that was recently submitted to GenBank from the Broad Institute. Comparison of genomic and mRNA sequences revealed six coding and one 5'-terminal non-coding exon in the equine *RNASEL *gene. This exonic composition is similar to that of a number of other mammalian *RNASEL *genes. However, two and three 5'-terminal non-coding exons were found in the chicken and mouse *RNASEL *genes, respectively. The coding vertebrate *RNASEL *exons were designated A through F. Comparison of the genomic and mRNA sequences of vertebrate *RNASEL *genes revealed significant length variation in both the 5'- (1402–1510 bp) and 3'-terminal (130–187 bp) coding exons (Table 5).

### SNP identification in the horse *RNASEL *gene

After identification of the equine *RNASEL *introns, exon-specific genomic primers were designed (Table 1). Exon-specific sequencing of DNA from the screening population identified 31 SNPs within the *RNASEL *gene (Table 6). Of the 10 non-coding polymorphisms, one was within the second intron and the others were located in the 5' and 3' UTRs. Seventeen of the 31 SNPs were located within the ankyrin repeat-encoding exon 2, 13 of which are non-synonymous, with 10 resulting in substitutions of amino acids with different properties. Three non-synonymous polymorphisms were identified within exons 3 and 5. The remaining exons, including the non-coding exon 1 were invariant among these horses. The amino acids encoded by the major allele of 11 of the 16 non-synonymous mutations were identical to the corresponding human RNase L amino acid [UniProtKB: Q05823]. Using MOTIF Search [45] to identify putative transcription factor binding motifs in the TRANSFAC database, the promoter SNP was found to be located within a potential cAMP-response element binding site (Score: 90) upstream of the first exon. Haplotypes were assembled in the same manner as for the equine *OAS1 *gene. The best reconstruction from Phase analysis produced 10 haplotypes among the 31 verified diallelic SNPs with minor allele frequencies = 0.08 (Table 3). As with *OAS1*, only good quality, unambiguous resequencing data were used for the haplotype analysis.

**Table 6:** Equine *RNASEL *single nucleotide polymorphisms

**Region**	**Accession **EF070193	**Alleles**	**Residue**	**Amino Acid Type**	**Frequency**	**Polymorphism**
-	143	C	-	-	0.12	Transversion
		G			0.88	
5' UTR	1857	A	-	-	0.55	Transversion
		C			0.45	
Exon 2	1991	C	27His	Basic Polar	0.54	Transition
		T	27Tyr	Uncharged Polar	0.46	
Exon 2	2020	C	36Gly	Uncharged Polar	0.92	Transition
		T	36Gly		0.08	
Exon 2	2021	G	37Asp	Acidic Polar	0.69	Transversion
		T	37Tyr	Uncharged Polar	0.31	
Exon 2	2118	A	69Asn	Uncharged Polar	0.29	Transversion
		C	69Thr	Uncharged Polar	0.71	
Exon 2	2121	A	70Tyr	Uncharged Polar	0.92	Transition
		G	70Cys	Uncharged Polar	0.08	
Exon 2	2316	A	135Lys	Basic Polar	0.75	Transversion
		C	135Thr	Uncharged Polar	0.25	
Exon 2	2332	A	140Ala	Nonpolar	0.35	Transition
		G	140Ala		0.65	
Exon 2	2374	G	154Arg	Basic Polar	0.83	Transversion
		T	154Ser	Uncharged Polar	0.17	
Exon 2	2635	A	241Thr	Uncharged Polar	0.21	Transition
		G	241Thr		0.79	
Exon 2	2680	C	256Ser	Uncharged Polar	0.43	Transversion
		G	256Ser		0.57	
Exon 2	2771	A	287Lys	Basic Polar	0.57	Transition
		G	287Glu	Acidic Polar	0.43	
Exon 2	3144	A	411Asn	Uncharged Polar	0.19	Transition
		G	411Ser	Uncharged Polar	0.81	
Exon 2	3152	C	414Arg	Basic Polar	0.81	Transition
		T	414Cys	Uncharged Polar	0.19	
Exon 2	3281	A	457Lys	Basic Polar	0.19	Transition
		G	457Glu	Acidic Polar	0.81	
Exon 2	3301	A	463Lys	Basic Polar	0.19	Transversion
		C	463Asn	Uncharged Polar	0.81	
Exon 2	3311	C	467Pro	Nonpolar	0.19	Transition
		T	467Ser	Uncharged Polar	0.81	
Exon 2	3372	A	487Gln	Uncharged Polar	0.58	Transition
		G	487Arg	Basic Polar	0.42	
Intron 2	3404	A	-	-	0.19	Transition
		G			0.81	
Exon 3	5108	A	513Lys	Basic Polar	0.11	Transition
		G	513Glu	Acidic Polar	0.89	
Exon 3	5111	C	514Pro	Nonpolar	0.82	Transition
		T	514Ser	Uncharged Polar	0.18	
Exon 5	7314	A	598Asn	Uncharged Polar	0.87	Transition
		G	598Asp	Acidic Polar	0.13	
3' UTR	9994	C	-	-	0.15	Transversion
		G			0.85	
3' UTR	9999	A	-	-	0.88	Transversion
		T			0.12	
3' UTR	10247	C	-	-	0.31	Transition
		T			0.69	
3' UTR	10914	C	-	-	0.38	Transition
		T			0.62	
3' UTR	11105	C	-	-	0.88	Transition
		T			0.12	
3' UTR	11146	C	-	-	0.35	Transition
		T			0.65	
3' UTR	11184	C	-	-	0.35	Transition
		T			0.65	
3' UTR	11228	C	-	-	0.83	Transition
		T			0.17	

SNPs identified from sequencing *RNASEL *exons from 13 equine individuals, CHORI BAC 159N12 and the reference transcript sequence. Positions are identified from the genomic consensus sequence submitted to GenBank [GenBank: EF070193].

Identifying single nucleotide polymorphisms by sequencing DNA from multiple individuals enhances the possibility of artifacts either from PCR or sequencing error. The 64 SNPs identified from the equine *OAS1 *and *RNASEL *genes were considered valid if each allele was identified in at least two individuals. Eight additional SNPs were identified but could not be verified in more than one individual (minor allele frequency < 0.08). Within the 3,864 and 5,406 base pairs re-sequenced during the SNP identification for *OAS1 *and *RNASEL*, respectively, equine *OAS1 *contained an average of one polymorphism per 117 bases, while equine *RNASEL *averaged one polymorphism per 174 bases.

## Discussion

Sequence characterization of the horse *OAS1 *gene in CHORI-241 BAC 100I10 enabled a partial genomic sequence assembly [GenBank: DQ536887] and comparison among multiple equine individuals. We identified 2 polymorphic microsatellites and 33 single nucleotide polymorphisms from a group of 13 individuals and CHORI-241 BAC 100I10 (Table 2). In an attempt to identify potential structural and/or functional consequences of the coding non-synonymous SNPs, each was analyzed using PolyPhen software [46-48]. Each polymorphic variant identified in equine OAS1 was predicted to cause benign effects at their respective residue position. However, the single mutation resulting in an Arg209Cys substitution may significantly change OAS1 enzymatic activity. Arg209 in the equine OAS1 protein corresponds to Arg544 in the human OAS2 protein, which is located in the donor binding domain. Substitution of Arg544 with either Ala or Tyr significantly decreased enzymatic activity of the OAS2 protein [49]. In addition, the equine *OAS1 *promoter SNP at position 4531 is located in an interferon stimulating response element [29]. Inactivation of this regulatory element by a single nucleotide substitution may alter expression of the equine *OAS1 *gene.

*RNASEL *enzymatic activity was previously reported in reptiles, birds, and mammals [50]. However, no *RNASEL *genes have been found for amphibians or fishes to date. Interestingly, the same classes of vertebrates also do not have OAS genes[51].

The horse *RNASEL *gene was FISH mapped to chromosomal location ECA5p17-p16. Orthologous genes are located on primate chromosome 1 (human, chimpanzee and rhesus macaque), cattle chromosome 16, dog chromosome 7, mouse chromosome 1, rat chromosome 13 and chicken chromosome 8 [52]. Using comparative chromosome painting (Zoo-FISH), similarities between human chromosome 1 and horse chromosome 5 [53], mouse chromosome 1, rat chromosome 13 [54], dog chromosome 7 [55,56] and cattle chromosome 16 [57] were previously established. Our results further confirm the conservation of *RNASEL*-containing syntenic chromosomal segments in horses.

Thirty one SNPs were identified for equine *RNASEL *(Table 6). Interestingly, all but three of the 20 coding SNPs identified are located within exon 2. The RNase L protein contains 9 N-terminal ankyrin repeats responsible for binding 2-5A molecules that are essential for activation [27]. Exon 2 of the human *RNASEL *gene encodes the entire ankyrin repeat region (amino acid 24 to 329). The high frequency of non-synonymous polymorphisms within exon 2 suggests that a single SNP or haplotype could ablate 2-5A binding and/or other RNase L interactions. As well, the SNP identified within the promoter upstream of the first exon is located within a potential cAMP-response element binding site. Mutations within this promoter element have been shown to affect gene expression [58-60]. PolyPhen analysis was also conducted on the non-synonymous coding SNPs identified within equine RNASEL. All but 4 of the RNase L SNPs were predicted to have benign effects. However, the SNP at residue 287 was predicted to change hydrophobicity at a buried site within the RNase L protein and the effect of this on protein function is unknown. The predictions provided by PolyPhen analysis are based on functional effects identified using human nsSNPs and may differ for the horse RNase L. Four SNPs within the ankyrin repeat region in exon 2 (residues 414, 463, 467 and 487) were predicted to have a negative effect on function. These data support our hypothesis that a single SNP or haplotype could affect 2-5A binding within the equine RNase L ankyrin repeats.

A number of SNPs were detected within the 3'UTR region of the equine *RNASEL *gene. Of the eight SNPs found within this region, six result in transitions. The 3'UTR regions of mRNAs contain regulatory regions capable of protein and microRNA binding that control mRNA stability, translation and localization. A simple analysis of octamer motifs containing equine 3' UTR SNPs identified SNP 10247 as being within a human miRNA target site [61]. If this target site is conserved in horses, this SNP could significantly affect the synthesis of RNase L. However, this particular octamer motif was not found in human or rodent *RNASEL *3'UTRs. Furthermore, cross-species sequence comparison using mVISTA[62,63] also revealed no significant longer range conservation in this region between species (data not shown).

Genotype analysis using PHASE v2.1 [40,41] identified 15 and 10 haplotypes among equine *OAS1 *and *RNASEL *genes, respectively, and suggested the existence of haplotype blocks spanning most of each gene (Table 3). Even if efforts to show an association between viral-induced disease susceptibility and *OAS1 *and/or *RNASEL *SNPs are successful, it may prove difficult to unambiguously identify a single causal SNP because of potential linkage disequilibrium at these loci. As determined from our screening population, a single haplotype occurred more frequently than any other, with a frequency of 0.19 and 0.23 in *OAS1 *and *RNASEL*, respectively (Table 3).

The frequency of SNP identification in this study in two equine genes was high considering the previously estimated equine SNP frequency of 1 per 1500 bp [64]. In dogs, the estimated SNP rate is ~1 per 1600 bp (based on entire genome re-sequencing), but a higher frequency of ~1 per 900 bp was estimated between breeds [65]. Re-sequencing of specific genes in several breeds of the domestic dog identified polymorphisms at frequencies comparable to our estimates, with 1 SNP per ~250–330 bp [S. Canterbury, personal communication]. Furthermore, re-sequencing within an Elk (*Cervus elaphus nelsoni*) putative promoter region, which is highly conserved between mule deer, cow and sheep, detected an average SNP frequency of 1 per 69 bp [unpublished data].

The microsatellite identified within the promoter region in this study may also alter expression of the equine *OAS1 *gene. The alleles observed to date indicate that dinucleotide repeat lengths of 9 and 18 may represent the major alleles at this locus. The over-representation of these alleles may be due to the fact that they correspond to one complete rotation of the DNA helix. If this microsatellite separates cis-regulatory elements, alterations in its length could affect the binding of transcriptional regulators to these elements and significantly alter gene expression [66-71]. In support of this hypothesis, there is a high degree of conservation between human and horse *OAS1 *promoters in the regions flanking the microsatellite (Figure 1). As well, recent micro-array data provide evidence of an inverse relationship between gene expression and dinucleotide microsatellite length, supporting the significantly higher frequency with which we identified the (GT)_9 _allele within the individuals screened[66]

## Conclusion

We report the genomic sequences of the equine *OAS1 *and *RNASEL *genes and identify 64 single nucleotide polymorphisms and 2 polymorphic microsatellites in these genes. On the basis of the allelic variants characterized, we conclude that a number of these are plausible candidates for regulatory or structural mutations which may influence transcription or enzymatic activity of OAS1 and RNase L proteins. Also, *RNASEL *cDNA sequences were determined for 8 mammals and utilized in a phylogenetic analysis. The chromosomal location of the RNASEL gene was assigned by FISH to ECA5p17-p16.

## Methods

### *RNASEL *cDNA and FISH

Preparation of horse cDNA was described previously [32]. Partial *RNASEL *sequences were extended using a DNA Walking SpeedUp Kit (Seegene USA, Del Mar, CA) according to the manufacturer's protocol. Four high-density filters for segment 1 of the CHORI-241 equine genomic BAC library were purchased from the Children's Hospital Oakland Research Institute (CHORI), Oakland, CA. These filters were screened using a P^32^-labeled equine *RNASEL *cDNA probe according to the supplier's protocol. Two positive equine BAC clones were purchased from CHORI. Each of these BAC clones was grown individually in 500 mL of LB media. BAC DNA was isolated using the NucleoBond BAC Maxi Kit (BD Biosciences Clontech, Palo Alto, CA) and used as the template for direct partial sequencing with a BigDye terminator v1.1 Cycle Sequencing Kit on an ABI 3100 Genetic Analyzer according to the manufacturer's recommendations. DNA from equine BAC clones 108P15 and 189I19 was FISH mapped as described previously [67]. International cytogenetic nomenclature of the domestic horse [68] was used to identify individual horse chromosomes.

The njtree program was used to construct a phylogenetic tree as described previously [51] and tree topology was inferred by the Neighbor-Joining algorithm. The bootstrap algorithm with 1000 replications was used to estimate the confidence of each node. The njtree program is available upon request.

### Construction of subclone library

BAC clone 100I10 was isolated from segment 1 of the CHORI-241 equine BAC library at Texas A&M University and confirmed by PCR as containing *OAS1*. The colony-isolated clone was cultured and BAC DNA was isolated by standard alkaline/lysis miniprep using Millipore Solutions and treated with Plasmid-Safe ATP-dependent DNAse (Epicentre, Madison, WI). BAC DNA was fragmented using a HydroShear^® ^DNA Shearing Device (GeneMachine, San Carlos, CA) at Speed Code 8 for an estimated fragment size of 2.5 kb. The fragmented product was analyzed by agarose gel electrophoresis stained with ethidium bromide and gel extracted using the QIAquick Gel Extraction Kit (Qiagen, Valencia, CA). Extractions were eluted in water according to the manufacturer's protocol. Purified fragments were cloned into vector pCR^® ^4Blunt-TOPO^® ^using the TOPO^® ^Shotgun Subcloning Kit (Invitrogen, Carlsbad, CA) following the manufacturer's protocol. Ligation reactions were incubated 30 minutes at room temperature and electroporated into *E. coli*. Colonies were screened for lack of β-galactosidase activity and selected for ampicillin resistance on LB-agarose plates containing 50 μg/mL ampicillin. White colonies were cultured and screened for appropriate insert size by PCR using vector-sequence M13 primer sites flanking the cloned insert, prior to sequencing.

### Sequencing of clones

Individual *OAS1 *inserts were amplified directly from individual colonies by PCR using vector-sequence M13 primer sites flanking the cloned insert. Amplification products were purified by centrifugation with the PSI-Clone PCR 96 kit (Princeton Separations, Adelphia, NJ) according to manufacturer's protocol. Purified products were sequenced in separate reactions with each M13 primer using a cycle sequence of 96C, 10 sec; 50C, 5 sec; 60C, 4 min with BigDye^® ^Terminator Mix v1.1 (Applied Biosystems, Foster City, CA). Sequencing reactions were analyzed using an ABI Prism 3100 Genetic Analyzer (Applied Biosystems, Foster City, CA).

Primers were designed to amplify the immediate promoter and exons of *OAS1 *and *RNASEL *genes from 13 individual horses by PCR (Table 1). Sequencing was carried out in the same manner as used for the library subclones. Sequences obtained were compared between individuals to identify SNPs within the amplified regions.

### Sequence analysis and contig assembly

Sequences were assembled and analyzed using Phrap assembly software [33,34,37] and viewed with the Consed visualization tool [35-37]. Contig and singleton reads were assembled by scaffolding onto the human genome using BLASTN [69-71].

Additional sequences were added to the assembly data and re-analyzed with Phrap and BLAST until the consensus sequence spanned the genes from the promoter to the 3' UTR. The genomic equine consensus sequence was confirmed using data from the Equine Genome Sequencing Project (2x) [38] and intron/exon boundaries were assigned by local alignment to the full-length equine *OAS1 *[GenBank: AY321355] and *RNASEL *[GenBank: DQ497159] cDNAs. The equine genomic sequences of *OAS1 *and *RNASEL *were submitted to GenBank and assigned the accession numbers DQ536887 and EF070193, respectively.

### Genotyping population

Blood samples were collected at the Texas A&M University Equestrian Center in accordance with ethical standards. The sampled set used for screening consisted of 13 horses, including 10 geldings/stallions and 3 mares, ranging in age from 21 months to 20 years. Breeds represented include American Quarter Horse (9), Arabian (1), American Paint Horse (1), Appaloosa (1) and Thoroughbred (1).

## Authors' contributions

JJR and DLA provided the *OAS1 *and *RNASEL *genomic sequence and assembled the haplotypes for both genes. AAP and MAB contributed all of the cDNA sequences for RNASEL. AAZ completed the phylogeny analysis. TLL completed the FISH analysis for RNASEL. JJR, AAP, MAB and DLA contributed to the identification of polymorphisms and helped to draft the manuscript. All authors read and approved the final manuscript.
